# Supplementary figures and images for: Requirement of a functional ion channel for Sindbis virus glycoprotein transport, CPV-II formation, and efficient virus budding
Source: PLoS Pathog. 2022 Oct 3;18(10):e1010892. doi: 10.1371/journal.ppat.1010892 (PMC9560593; doi:10.1371/journal.ppat.1010892)

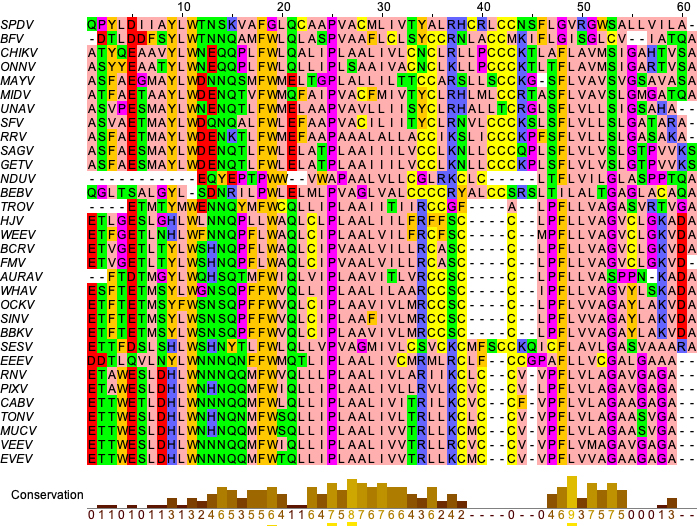

Supplement: S1 Fig — Alignment files were generated using CLUSTAL omega and sequence alignments were viewed using Jalview. (JPG) [file ppat.1010892.s001.jpg]

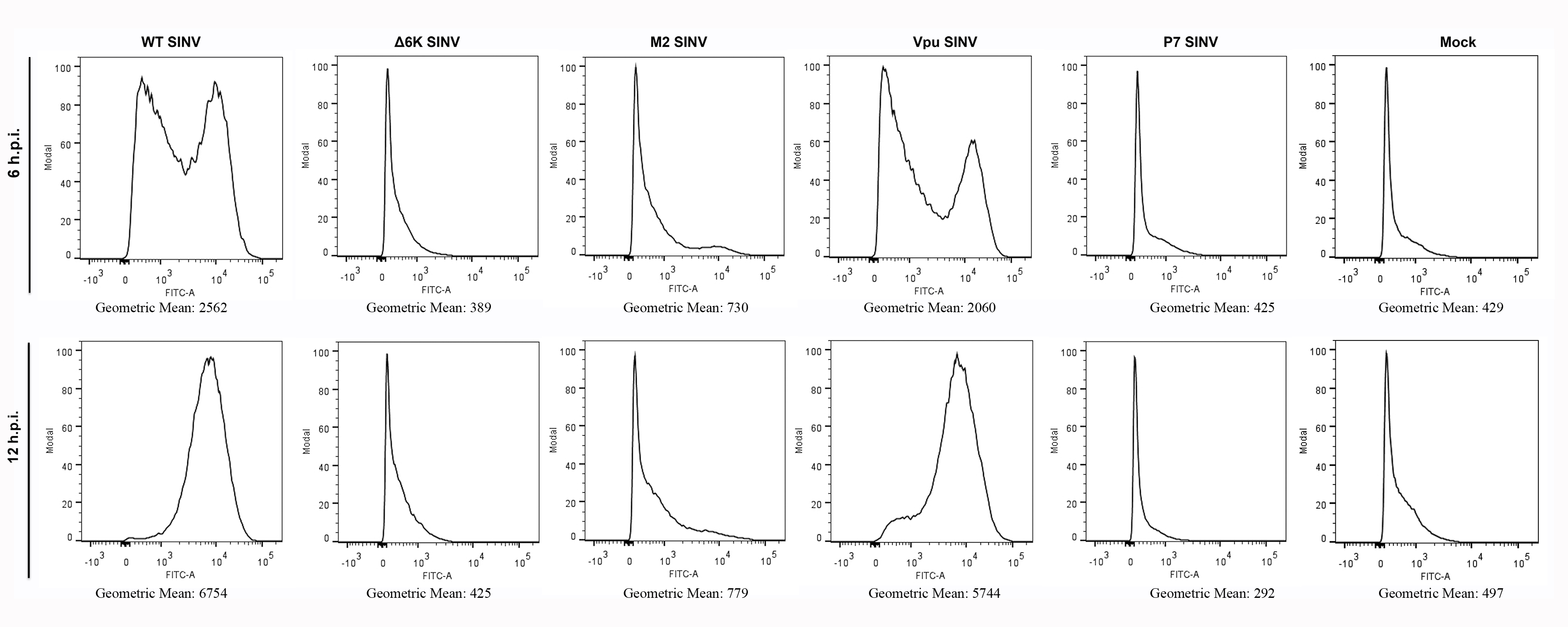

Supplement: S2 Fig — Representative flow charts of cells infected with WT or 6K mutant viruses at an MOI of 5. Cells were incubated with a monoclonal anti-E2 antibody followed by staining with FITC secondary antibody. (JPG) [file ppat.1010892.s002.jpg]

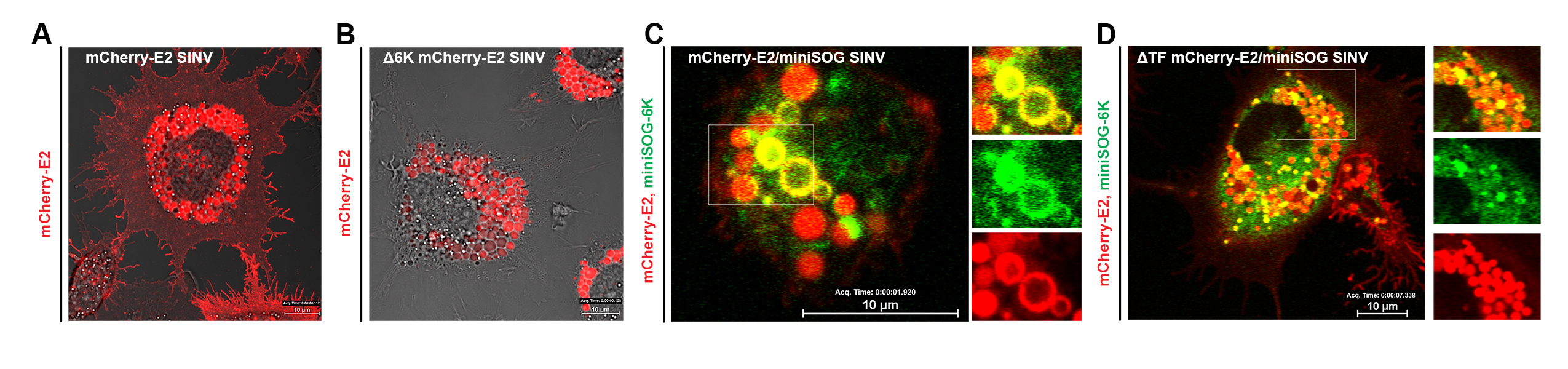

Supplement: S3 Fig — (A-B). Representative images of C6/36 cells infected with mCherry-E2 SINV (red) or Δ6K mCherry-E2 SINV (red) and imaged at 24 hpi. (C-D). Representative images of C6/36 cells infected with the dual-labeled mCherry-E2/miniSOG SINV (red and green) (C) or the ΔTF mCherry-E2/miniSOG SINV (red and green) (D) and imaged at 24 hpi. (JPG) [file ppat.1010892.s003.jpg]

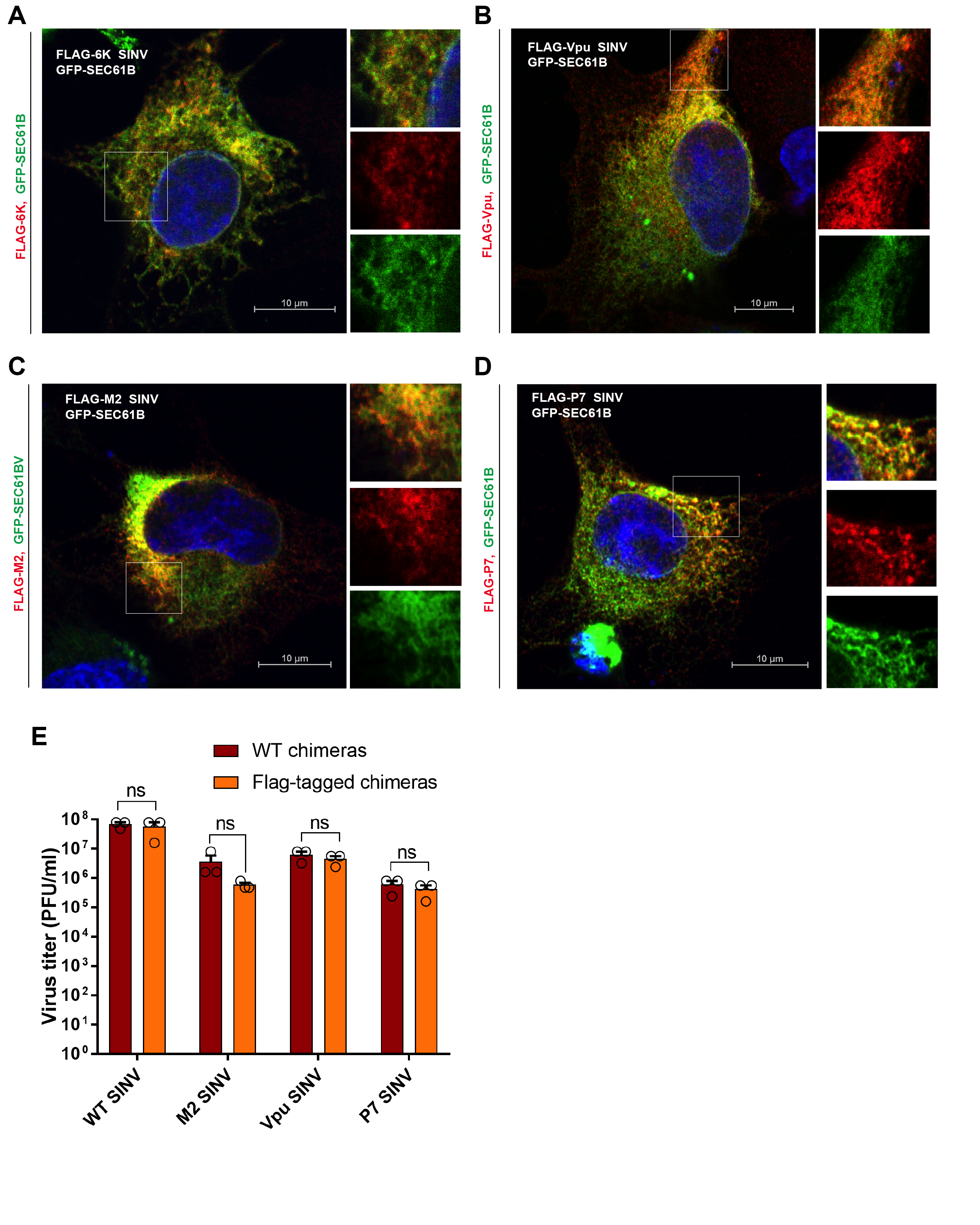

Supplement: S4 Fig — IF analysis using anti-FLAG antibody of permeabilized BHK-15 cells transfected with mEmerald-SEC61B-C1 and infected with (A) FLAG-6K SINV, (B) FLAG-Vpu SINV, (C) FLAG-M2 SINV, or (D) FLAG-P7 SINV. Cells were fixed at 12 hpi. (E) Plaque assays of WT and Flag-tagged viruses. Data shown are from three independent experiments. Error bars indicate standard error of mean (SEM). Significance was determined by multiple unpaired t-tests of data. p Values were considered significant when p < 0.05 (*), p < 0.01 (**), p < 0.001(***), and p < 0.0001(****). ns indicates “not significant”. (JPG) [file ppat.1010892.s004.jpg]

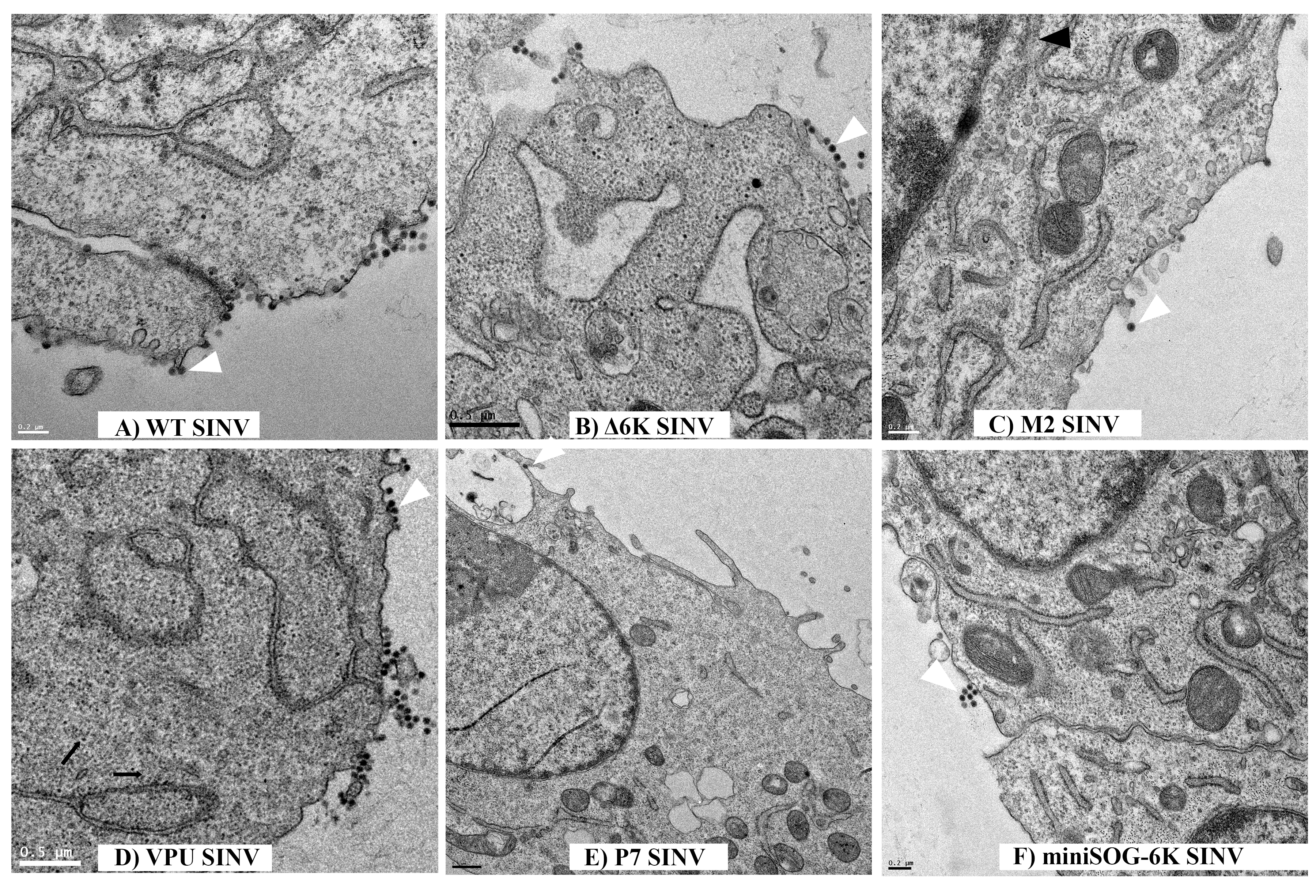

Supplement: S5 Fig — TEM analysis of BHK-15 cells. Cells were infected with WT SINV (A), Δ6K SINV (B), M2 SINV (C), Vpu SINV (D), P7 SINV (E), or miniSOG-6K SINV (F), and fixed at 12 hpi. White arrowheads indicate budding viruses. (JPG) [file ppat.1010892.s005.jpg]
