# Supplementary material for: Requirement of a functional ion channel for Sindbis virus glycoprotein transport, CPV-II formation, and efficient virus budding
Source: PLoS Pathog. 2022 Oct 3;18(10):e1010892. doi: 10.1371/journal.ppat.1010892 (PMC9560593; doi:10.1371/journal.ppat.1010892)
Supplement: S1 Table — (DOCX) [file ppat.1010892.s013.docx]

Supplementary table 1

| Mutant | Forward Primer | Reverse primer |
| --- | --- | --- |
| Δ6K SINV | AAGGTAGACGCCTACGAACATGCGACC | GTAGGCGTCTACCTTGAACGTTTCAGCATTGGCCGACCTAAC |
| ΔTF SINV | CTGCCGTTCCTCGTGGTTGCCGGCGCCTACCTGGGCG | AACCACGAGGAACGGCAGGCAGCAGGAGCAGCAGCATTAG |
| M2 SINV | TTGCACTTGATACTCTGGATTCTTGACCGTCTGTTCCTGCCTTTTTTAGTGGTTGCCGG | GAGTATCAAGTGCAAGATGCCGATAATGGAGGCCGCGACAACCAGAGGATCGGAAGACGTTTCAGCATTGGCCGACCTAAC |
| Vpu SINV | TTATCATAGCCATCGTCGTATGGTCCATCGTTATCATAGAATATCGCAAGATCCTGCCTTTTTTAGTGGTTGCCGGCGCC | CGATGGCTATGATAATAGCTACGACGAGTGCTACGATTGCGACGATAGGGATAGGTTGGGTCTCGGTGAACGTTTCAGCATTGGC |
| P7 SINV | TCTTTGTTGCCGCGTGGTATATTAAAGGAAGAGTCGTTCCGCTTGCAACGTACTCGCTAACAGGACTCTGGTCATTCAGTCTGTTACTGTTAGCTCTCCCTCAGCAAGCATACGCATATAAGGTAGACGCCTACGAACATGCGACC | ACGCGGCAACAAAGAATATTACGAAGTACAGAAATCCGTTACAAGAAGCCGCGCTCGCTGCGTGCAGTATGACTAACTTCTCCAAGGCTTCAGCATTGGCCGACCTAACGCAGC |
| miniSOG-6K SINV | ATGGAGAAAAGTTTCGTGATAACTG | TCCATCCAGCTGCACTCCGATGAAATACTGAAGTTCACCCTTCTGGTCCCTCATGGGC |
| miniSOG-6K Overlap SINV | GTGCAGCTGGATGGATTCACCGAGACCATGAGTTACTTG | GAAACTTTTCTCCATCGTTTCAGCATTGGCCGACCTAAC |
| FLAG-6K SINV | AAAGATGATGACGACAAAACGTTCACCGAGAC | CTTATCGTCATCGTCCTTGTAGTCCGCCAGGTAGGCGCCGGCAACCAC |
| FLAG-M2 SINV | AAGATGATGACGACAAAACGTCTTCCGATCCTCTGGTTGTCGCG | CTTATCGTCATCGTCCTTGTAGTCCGCCAGGTAGGCGCCGGCAACCAC |
| FLAG-Vpu SINV | AAGATGATGACGACAAAACCCAACCTATCCCTATCGTCGCAATCG | CTTATCGTCATCGTCCTTGTAGTCCGCCAGGTAGGCGCCGGCAACCAC |
| FLAG-P7 SINV | AAGATGATGACGACAAAGCCTTGGAGAAGTTAGTCATACTGCACG | CTTATCGTCATCGTCCTTGTAGTCCGCCAGGTAGGCGCCGGCAACCAC |
