# Supplementary material for: Requirement of a functional ion channel for Sindbis virus glycoprotein transport, CPV-II formation, and efficient virus budding
Source: PLoS Pathog. 2022 Oct 3;18(10):e1010892. doi: 10.1371/journal.ppat.1010892 (PMC9560593; doi:10.1371/journal.ppat.1010892)
Supplement: Attachment — Submitted filename: Cover letter with response-8-8.docx [file ppat.1010892.s014.docx]

Department of Biochemistry

and Molecular Biology

Eberly College of Science

The Pennsylvania State University

Paul M. Althouse Laboratory

University Park PA 16802-4500

814-865-5497

Fax: 814-863-7024

Dear Editor,	August 7, 2022

RE: (PPATHOGENS-D-22-00829)

We are pleased to submit our revised manuscript entitled " **Requirement of a functional ion channel for Sindbis virus glycoprotein transport, CPV-II formation, and efficient virus budding."** We thank the editors and the reviewers for acknowledging the high interest in the topic and the constructive comments on our previous resubmission of the manuscript. In the revised manuscript, we have incorporated new experiments and provided new images with improved quality. We have restructured our manuscript addressing the reviewers' major concerns and highlighting our manuscript's novelty. We have provided new supplementary data in the revised manuscript that support our conclusions. However, due to the lack of reagents and expertise, we have not been able to perform some of the studies suggested by the reviewers. These include new electron microscopy experiments and protein expression and purification of bacterially expressed membrane glycoproteins E1, E2, and 6K to determine the interaction of 6K. We hope that the revised manuscript with new supplementary information clearly explains our latest findings on the role of 6K protein in alphavirus assembly. While we agree that there are several further studies possible to understand the functional role of 6K protein mechanistically, as explained in response to the reviewers, some of these experiments are beyond the scope of this manuscript. We believe that addressing the concerns of the editors and reviewers has significantly improved the quality and strengthened our manuscript. We hope that this revised manuscript is now suitable for consideration for publication by PLOS Pathogens.

Please find below our point-by-point responses to the reviewers' comments. The italicized blue font represents the response.

Sincerely,

Joyce Jose, Ph.D.,

Assistant Professor of Biochemistry & Molecular Biology

The Pennsylvania State University

University Park PA 16802-4500

email jxj321@psu.edu

Elmasri et al find the 6K protein in Sindbis can be replaced by the Vpu protein from HIV, and less so by M2 and p7 from Influenza and HCV, respectively. They go on to propose that 6K and E2 colocalize in the Golgi and these proteins together are responsible for CPV II formation, promoting E2-E1 glycoprotein transport. Finally, they conclude that the ion channel activity of 6K is what is driving CPV II formation and glycoprotein transport. Very little work has been done on 6K and its role as an ion channel, the scientific impact of this research is high. However, as both the reviewers and myself point out, there are some concerns with the conclusions drawn from the results.

**The major issues addressed by more than one reviewer:**

1. A majority of the conclusions are based on correlation and no evidence of a direct interaction between 6K and E2. Some suggestions include complementation experiments--will expression of E2 and 6K be enough to form CPV II? Do the chimera viruses also form CPV IIs? Any ideas how ionic flux is mediating CPV II formation and why are these vesicles more likely to transport glycoproteins to the plasma membrane? More suggestions are in the reviewer comments. In addition, better images including more quantification, for both IF colocalization and TEM CPV II formation would strengthen the manuscript.

*We thank the editor and the reviewers for these suggestions. Determining the minimum requirement for CPV-II formation will be an important aspect of the alphavirus life cycle. Our manuscript provides the importance of 6K in the formation of CPV-II. We have provided new figures with improved quality to accurately show the colocalization of proteins, as demonstrated below. Although highly informative, we suggest some of the suggested experiments are outside the scope of this manuscript.*

2. Figure 3 is not clear. nor is it complete. The text, figure, and legend indicated different constructs being used making drawing conclusions difficult. More analysis on the trafficking of E2 to the plasma membrane is necessary. For example, is E1 produced? does it transit to the plasma membrane? The western blot doesn't show if E1 is produced (side note: The western is with anti-E2 yet Capsid is on the blot).

*Fig. R1. Subcellular localization of E1 and E2. IF analysis of permeabilized BHK-15 cells infected with WT SINV or Δ6K SINV at 6 or 12 h.p.i. stained using primary anti-E2 monoclonal antibody and (Green) anti-E1 polyclonal antibody (Red).*

*We thank the reviewer for these comments. We have provided answers to these questions and have replaced figure 3 with a revised figure that has a plasma membrane marker showing the trafficking of mCherry-E2. We have rewritten the figure legend and labels accordingly.*

*Two figures (Fig. R1, Fig R2) are added here to support our observations for the reviewers. We performed western blot analysis using an anti-E1 antibody and showed that E1 is produced and processed in the absence of 6K. We did immunofluorescence analyses using the anti-E1 antibody and showed that E1 trafficks to the plasma membrane along with E2 in the presence of 6K. Whereas in the absence of 6K, E1 and E2 trafficking to the plasma membrane is severely inhibited at early time points post-infection, and E1 and E2 proteins are detected at the plasma membrane only at later time points post-infection. Should the reviewers suggest these images are required in the manuscript, these figures can be added as supplementary figures. As the reviewers noted, anti-E1 and anti-E2 polyclonal antibodies also bind capsid protein nonspecifically. The monoclonal anti-E2 antibody used in the IFA and flow cytometry does not have the background. However, this antibody does not detect E2 in western blot analysis.* *Therefore due to the lack of reagents, we are unable to generate a western blot with no background.*

3. Why is Vpu able to replace 6K so efficiently and M2 and p7 cannot? Some further discussion is warranted, even structural predictions of the different putative ion channel proteins. It should also be addressed that recent work has shown 6K might not be two transmembrane helices but rather a single pass TM helix, similar to Vpu. Does the coronavirus E protein substitute for 6K?

*Fig. R2. Western analysis of wild-type SINV and ∆6K SINV. BHK-15 cells were electroporated with in-vitro transcribed RNA of wild-type SINV and ∆6K SINV in 6-well plates. Infected cells were lysed using lysis buffer [25mM Tris-HCl pH 7.5, 150mM NaCl, 1% Triton-X 100, 5mM 2-mercaptoethenol, 1mM PMSF]. Cell lysates were harvested at 12 hours and 24 hours post electroporation and run on a 4-20% precast SDS-PAGE gel (Bio-Rad). Proteins were transferred onto a nitrocellulose membrane and probed with mouse monoclonal anti-actin antibody and rabbit polyclonal antibodies against SINV E1 and E2 proteins. Infrared labeled secondary antibodies (goat anti-mouse 680 and goat anti-rabbit 800) were used for detection. Blots were imaged using an Odyssey CLx Near-Infrared Imaging System and analyzed using Licor Image Studio software.*

cells were electroporated with in-vitro transcribed RNA of wild-type SINV and ∆6K SINV in 6-well plates. Infected cells were lysed using lysis buffer [25mM Tris-HCl pH 7.5, 150mM NaCl, 1% Triton-X 100, 5mM 2-mercaptoethenol, 1mM PMSF]. Cell lysates were harvested at 12 hours and 24 hours post electroporation and run on a 4-20% precast SDS-PAGE gel (Bio-Rad). Proteins were transferred onto a nitrocellulose membrane and probed with mouse monoclonal anti-actin antibody and rabbit polyclonal antibodies against SINV E1 and E2 proteins. Infrared labeled secondary antibodies (goat anti-mouse 680 and goat anti-rabbit 800) were used for detection. Blots were imaged using Odyssey CLx Near-Infrared Imaging System and analyzed using Licor Image Studio software.

*We have modified the discussion to include the effects of Vpu. We have incorporated the recent work on 6K topology and added a discussion of topology. Since there are several viral ion channels, we will compare the effect of these channels, including coronavirus E protein, in replacing alphavirus 6K in our future studies. We believe these experiments are not needed to support our results in this manuscript.*

**Reviewer's Responses to Questions**

**Part I – Summary**

Please use this section to discuss strengths/weaknesses of study, novelty/significance, general execution and scholarship.

**Reviewer #1:**

In this study, Elmarsi et al., look to understand the function of the Sindbis virus (SINV) 6K ion channel. They generate a SINV 6K deletion as well as chimeric viruses with known viral ion channels (very cool!). They show that 6K is required for virion production and glycoprotein trafficking to the plasma membrane. In addition, they found that Vpu can rescue virion production and E2 trafficking. Using live cell imaging, they show that the SINV E2 accumulates in the secretory pathway when 6K is absent, and that 6K localizes to the secretory pathway. TEM experiments show differences in CPV structures. Finally, they show that SINV is sensitive to the known Vpu inhibitor HMA suggesting that 6K is similar to Vpu and an antiviral target.

Together, this is a well-written study that provides new insight into SINV biology and begins to elucidate the role of 6K in SINV infection. However, there are several concerns regarding the study including experiments and the conclusions drawn.

Reviewer #2: The manuscript by Elmasri et al. analyzes a prominent member of the viroporin family – the 6K protein from alphaviruses. While the importance of 6K in the life cycle of alphaviruses is somewhat well established, the exact requirement for the ion channel activity is not understood. The authors have generated several chimeras by combining or replacing 6K in SINV by viroporins from other families like HCV p7, Influenza M2 and HIV Vpu, and tested their effect on virus particle production and infectivity, glycoprotein trafficking and other cellular phenomenon like formation of Type II CPVs. The comments and concerns regarding this work are as follows:

**Major points:**

1) The main findings of the study are a correlation between the availability of an effective ion channel activity, and the formation of type-II cytopathic vacuoles, which in turn correlates with spatiotemporally correct glycoprotein trafficking and virus particle release. The primary results are correlation based, and no direct interactions/effects are shown in the manuscript, which is a major flaw. For example, is there any direct interaction between the glycoprotein E2 and 6K/6K substitutes during infection or under in vitro conditions? This appears a very important point to establish the role of 6K in promoting glycoprotein trafficking.

*We thank the reviewer for these valuable comments. We agree with the reviewer that our observations are also based on the correlation between the presence of ion channels and CPV-II. Based on our results, we propose that the functional role of 6K as an ion channel is more important for the trafficking of E2. While a possible direct 6K-E2 interaction is a relevant mechanism, we have not performed experiments to establish these interactions. Interaction of 6K with E2 or E1 has not been determined either from a 3.5 Å cryo-EM structure of the Sindbis virus (1) or in vitro biochemical interaction studies. However, a genetic interaction has been reported based on revertant screening of chimeric viruses (2). As suggested by the reviewer, conducting an in vitro interactions study of 6K with E2 or E1 would require the expression and purification of three membrane proteins with TM helices. Although full-length 6K has been expressed and purified from bacteria, purification of E1 and E2 has not been reported. Similarly, pull-down studies have been reported from infected cells, and interactions of E1 or E2 with 6K using pull-down studies have not been reported. If only one protein is expressed, it would not be in a native heterodimer or in a trimeric spike form, and that interaction would not be indicative of the in vivo conditions. These experiments will be performed in the future when we have an established system of protein expression and purification.*

2) Also, what about the transport of E1? Do the constructs used in the study - E3-mCherry-E2-E1 and E3-mCherry-E2-6K/TF-E1 - have a cleavage site before E1? If so, the live cell imaging panel cannot ascertain where E1 is ending up since the tag is on E2.

*We thank the reviewer for their comment. In alphaviruses, heterodimerization and spike formation of E1 and E2 are critical for efficient envelope glycoprotein transport to the plasma membrane. Furthermore, when E1 and E2 are expressed individually, E1 is retained in the ER, and only a fraction of E2 is transported to the cell surface (3,4). Thus E1 gets transported along with E2 when expressed from the same polyproteins* (5–7)*. We have previously published the structure of mCherry tagged E2 SINV (8) clearly indicating the structural organization of mCherry protein on the virus surface. mCherry-E2 fluorescent protein-tagged constructs used in our manuscript have been validated in a previous paper (9). We have previously published the (10) requirements of E2 trafficking to the plasma membrane along with E1. Similarly, plasmids encoding structural polyproteins CP, E1, E2, E3, and 6K of chikungunya virus have been used for chikungunya VLP production (11), confirming that the expression constructs faithfully recapitulate E1/E2 glycoprotein trafficking to the plasma membrane as trimeric spikes, where the particle budding occurs. Additionally, our mammalian expression constructs are subcloned from the mCherry-E2 and delta 6K mCherry-E2 viruses (8). All expression constructs have been sequenced and confirmed, and we have not detected any issues with the lack of E1 expression from these plasmids. These constructs also have the original signalase cleavage site before E1. Based on the reviewer’s comments, we have now performed immunofluorescence analysis of cells infected with WT or ∆6K SINV at 6 and 12 h.p.i. Using primary antibodies against E1 and E2. Our results show that in the absence of 6K, E1 does not localize to the plasma membrane at early time points, similar to what we observed for E2. In addition, we performed western blot analysis on cells infected with WT or ∆6K SINV. Our results confirm that E1 is made in the absence of 6K. As seen in the IFA and western blots, the anti-E1 polyclonal antibody shows nonspecific binding and background. This proves that the E1 protein is cleaved and transported to the plasma membrane (Fig R1 and Fig R2).*

3) The authors find that Vpu closely mimics the functionality of 6K, followed by M2, and finally p7, which does not appear to be functionally close to 6K even though its membrane topology is closest to that of 6K. This anomaly is not probed further. In terms of sequence or predicted secondary or tertiary structural elements, is 6K closer to Vpu/M2 compared to p7?

*We thank the reviewer for their comment. There are no crystal structures available for 6K and the ion channel region of Vpu. In the absence of structure and biochemical analyses, we think it will be hard to predict the oligomeric stage of 6K. The membrane topologies have been proposed from biochemical and molecular genetic analyses. Based on previous studies and our results reported here, we propose that 6K and P7 have similar membrane topologies. However, our experiments show that, unlike 6K, P7 is mainly retained in the ER and does not localize to the Golgi apparatus. As discussed in the manuscript (lines 559-575) we have proposed that the difference in localization possibly explains why P7 is not as efficient as Vpu, which is localizing to the Golgi, in rescuing the function of 6K.*

4) Does overexpression of 6K or 6K+E2 in cells result in the formation of CPV-II, or does it only happen during SINV infection – this may point to the involvement of other viral proteins/RNA in the process?

*We thank the reviewer for this excellent suggestion. Thus far, CPV-II has only been reported from virus-infected cells (12,13). We are currently investigating the requirements for CPV-II formation, and these requirements are not within the scope of this manuscript.*

5) The manuscript mentions the differences in virus titre and specific infectivity of virus particles generated upon 6K deletion or 6K replacement with chimeric components. It will be great if this information is provided on the same scale (log vs fold decrease). A 2.8 fold decrease in specific infectivity of particles without 6K suggests that the defect is not only in release, but also in the assembled particles themselves. In this context, it is important to morphologically characterize SINV particles without 6K, or those containing 6K chimeras, in comparison to WT particles. This will add important information to the study.

*We agree with the reviewer that there might be a structural change in the virus in the absence of 6K (2,14,15). There are contradicting reports on whether there are structural changes in the absence of 6K (16). In this study, we are focusing on the defect in virus release, and we have provided sufficient supporting information to establish the functional role of 6K in alphavirus assembly and glycoprotein trafficking. Furthermore, our TEM analyses show budding particles that appear morphologically similar to WT SINV. Due to the vast number of experiments needed, some of which we currently lack the expertise, we have not explored the packaging of 6K or chimera into the virion. This would be an important follow-up study.*

6) Finally, there appears to be some difference in the behavior of alphaviruses with 6K deleted, as well as the susceptibility of 6K from different alphaviruses to drugs/inhibitors. How similar are the alphavirus 6K proteins in terms of sequence or structure?

*Based on the important comment of the reviewer, we have added a multiple sequence alignment of alphavirus 6K in the supplementary section. The sequence alignment shows that the first transmembrane helix is conserved among different alphaviruses, whereas the second helix is more variable.*

7) Along the same lines, it will be helpful to include the established roles of M2/Vpu/p7 in the life cycles of the respective viruses in the Introduction/Discussion for purposes of comparison to 6K.

*We thank the reviewer for this comment. We have included this information in the manuscript. The sections where we discuss the roles of these proteins are indicated (lines 105-116)*

**Minor point:**

The manuscript includes substantial experimental details in the Discussion section. The Results and Discussion sections should be reorganized for clarity.

*We have removed the experimental details from the discussion.*

Reviewer #3: The manuscript: Requirement of a functional ion channel for alphavirus glycoprotein transport, CPV-II formation, and efficient virus budding, but Elmasri, Negi, Kuhn, and Jose, uses a variety of imaging and chimeric virus methodologies to provide an in-depth characterization of the functional role of the viral 6K protein's ion pore activity in virion maturation and budding. While the pore forming activity of the alphavirus 6K protein, and its potential as an antiviral target has been well recognized within the alphavirus field, the specific stages in the viral lifecycle where ion channel activity acts have not been well characterized. Therefore, the study is important, and in general, the manuscript is well written, the experiments are well done, and the authors' conclusions are supported by the data. However, as noted below, there are some concerns that should be addressed to provide further support for the authors' conclusions, clarify discrepancies in the manuscript, or make it easier for the reader to follow.

**Part II – Major Issues: Key Experiments Required for Acceptance**

Please use this section to detail the key new experiments or modifications of existing experiments that should be absolutely required to validate study conclusions.

Generally, there should be no more than 3 such required experiments or major modifications for a "Major Revision" recommendation. If more than 3 experiments are necessary to validate the study conclusions, then you are encouraged to recommend "Reject".

Reviewer #1: 1. A major concern is that, as the authors state throughout the paper, functional studies of 6K have been completed in SFV. The authors have nicely compared their work to these previous works and shown that SINV is different than SFV. This finding is very cool and a comparative analysis of SFV in this paper would have been interesting. Nonetheless, as written, this paper is SINV specific and the authors should replace “alphavirus” throughout the paper, including the title, with SINV.

*We thank the reviewer for this suggestion. We have replaced alphavirus with SINV in the manuscript title and wherever it is relevant in the manuscript.*

2. Figure 1D shows a 1-step growth curve yet the y-axis is a “rate of virus release”. Can the authors also show the actual growth curve titers over time in the figure? I think it would be much easier to interpret this way. From the current figure, even the 6K deletion is pumping out 10K viruses/ml/hr. That seems like a lot still. Moreover, when comparing Figure 1 to Figure 7, WT, Vpu, and M2 viruses grow similarly in Figure 7 at MOI 0.1 (no drug) yet there are very different in Figure 1 (MOI = 5). Can the authors comment on these differences?

*We thank the reviewer for pointing out the differences between Fig 1 and 7. Virus one-step growth kinetics are indicated using the rate of virus release (pfu/ml/hour), as we have previously reported in several papers (10,12,17). Therefore, we are following the conventional way of representing growth kinetics. As shown in figure 1, there is a 4-log reduction in virus titer compared to the WT. The titers of mutants indicated in figure 1 are representative of actual virus release per hour. In figure 7, the pfu is not represented as virus release per hour, and it is the cumulative titer obtained after 8 hours, which explains the difference.*

3. Figure 3. A few points: A) The text states that these experiments are in the absence of capsid (Line 368-369) yet the figure and figure legend states that capsid is present. Can you confirm which is correct? These are important for your conclusion on line 373.

*We thank the reviewer for their comment and for noticing the discrepancy. We have now deleted CP from the labeling in Figures 3A and 3B as this experiment was done using E3-E1 constructs with no capsid.*

B) It is hard to see differences in Figure 3C. Can you use the membrane marker to make this clearer? Also, are there less filopodia in the delta6K virus? Could this be a phenotype?

*We thank the reviewer for these comments. We have used a membrane marker to help in visualizing the localization of E2 to the plasma membrane. In the revised manuscript, we have replaced panel 3 with a new figure. We agree with the reviewer's comment and note that we have observed fewer long extensions at earlier time points in the absence of 6K. This could be explained by previous studies indicating that the presence of CP and E2 at the plasma membrane is essential for the formation of long filopodial extensions* [*(1)*](https://doi.org/10.1371/journal.ppat.1006061)*.*

C) The protein processing is interesting. However, to conclude that the phenotype is not due to processing, I think showing complementation would strengthen your argument. Can you express 6K in trans (as you can with Vpu) and show that processing is restored? Or use the Vpu chimera virus?

*We thank the reviewer for their suggestion. We have tested the polyprotein processing in the absence of 6K at later time points post-infection and saw that the processing efficiency increases with time in the absence of 6K. We have included the figure here to show the processing over time (Fig. R2). This supports our conclusion that the delay in processing in the absence of 6K is due to a delay in glycoprotein trafficking to the late Golgi, where furin cleavage occurs. Furthermore, we have not observed any defects in glycoprotein trafficking or processing for the Vpu-chimera; hence we have not included that in the manuscript. It has also been reported for Semliki forest virus that the co-expression in trans of a WT 6K gene was unable to complement 6K or TF mutant viruses because the E1 signal peptide cannot operate in trans (18)*

*Fig. R3. Representative images of BHK-15 cells co-transfected with CP-E3-mCherry-E2-6K/TF-E1 (red) and mEmerald-SEC61B (green) (A) or mTagBFP2-SiT-N-15 (blue) (B). Regions of interest are marked and enlarged to the right as separate channels.*

4. All of your BHK experiments are completed in 12 hours. Could timing be an issue for the 6K mutant? Throughout the manuscript you use the word “Delayed” which implies time. If you waited longer, do you get more processing of the proteins in Figure 3D? (Similar to the one above)

*As addressed in question 3, we have observed an increase in the polyprotein processing in the absence of 6K at later time points (Fig. R2). If required, we can add this to the manuscript.).*

5. Figure 4: Here you use two tagged viruses that seem to behave very differently. In Figure 4E, the 6K and E2 are punctate and then in Figure 4F 6K and E2 are diffuse and seem to be on filopodia. While I don’t doubt 6K is in the secretory pathway, you may have different results for E2 (different compartments) depending on the system? Can you use the dual tagged virus with ER or Golgi markers? Also, what does the protein processing or virion production look like in the dual tagged virus? Is it restored?

*We thank the reviewer for their suggestion. The reason for the apparent difference in E2 localization is due to the different imaging planes (Z sections). We have now provided the reviewers with a representative Z-stack image showing the distribution of E2 in cells transfected with CP-E1 and infected with miniSOG-6K (Fig. R4). The image shows that E2 is localizing to vesicles and plasma membrane when expressed from expression plasmids, similar to its localization when expressed from the dual-labeled virus. We have also replaced Fig 4E with a better representative figure. Additionally, we have imaged our CP-E1 construct with ER and Golgi markers and found that, as expected, E2 localizes to both ER and Golgi. We have provided a figure for the reviewer to confirm the results (Fig R3). Imaging the dual labeled virus with markers has been challenging due to the rapid photobleaching of miniSOG when we used three lasers. We have used the dual labeled virus to verify the colocalization of E2 and 6K on vesicles, and our results have confirmed this colocalization. As we have mentioned in the manuscript, the incorporation of two fluorescent proteins on E2 and 6K negatively affects virus production. Therefore, we have used multiple experimental approaches, including the eukaryotic expression system, to verify the colocalization of 6K and E2 in the secretory pathway.*

6. The conclusion about CPV formation is interesting but I think more work needs to be done on this section. The TEM images are hard to see and interpret. I suggest zooming in and using some of your trans systems to prove your point. Regardless, this needs significant work for this submission.

*We thank the reviewers for these helpful comments. We have now provided a zoomed-in version of our TEM images for ease of interpretation (revised Fig. 6). We agree with the reviewer and believe that a significant amount of work is needed in this area. An elegant study has recently revealed how cryogenic electron tomography is utilized in visualizing the chikungunya virus assembly and budding in situ (19). Such studies using our mutants described in the manuscript will be highly useful in determining the functional role of 6K in alphavirus assembly. Although we are continuing the structural analysis of 6K, these suggested experiments will require expertise that is not currently established in our laboratory. Thus, we are unable to pursue these avenues for this manuscript and hoping to address these significant questions in a follow-up paper.*

*Fig. R4 Representative confocal Z-stack image generated using individual Z sections of BHK-15 cells transfected with CP-E3-mCherry-E2-6K/TF-E1 and infected with miniSOG-6K SINV. The region of interest is marked and shown as separate channels to the right.*

7. Your inhibitor experiment is awesome and an amazing tool to dissect the function of 6K in the absence of a deletion (which could have bigger issues). I think looking at protein processing, E2 trafficking, and CPV formation in the presence of HMA would add significantly to your conclusions.

*We thank the reviewer for their suggestion. Our goal was to show that inhibiting the ion channel activity affects virus titer. We agree that additional experiments, including structural studies, are required to determine the mechanism of HMA inhibition. Those studies are ongoing, and we believe that they are not required for the completion of this manuscript.*

**Reviewer #2:**

1) Analysis of direct binding of 6K to E2, under in vitro conditions or during co-expression in cells/infection

*We thank the reviewer for their suggestion. We have explained this above. The feasibility of this experiment is low because of the expression and purification of oligomeric membrane proteins. Because of the concerns regarding the time and feasibility, we have not added the suggested experiments to this manuscript.*

2) Same for 6K and E1

*We thank the reviewer for their suggestion. As explained above, we are planning to do these experiments in a future study.*

3) Formation of CPV-II in cells during overexpression of 6K/co-expression of 6K with viral proteins

*We thank the reviewer for this suggestion. We are planning to do this in a future study.*

4) Bioinformatics based comparative study of viroporins used in the manuscript

*We have incorporated the structural and functional role of different viroporins in the manuscript. Although bioinformatic analyses are useful, and several such studies have been published, the focus of this manuscript is the functional role of 6K in using imaging and virology experiments.*

5) Morphological analysis of SINV particles with 6K deleted/6K chimeras

*We thank the reviewer for their suggestion. We agree that this will be a highly interesting area of research, and we will address these aspects of 6K in a follow-up manuscript.*

Reviewer #3: 1) If ion channel activity is the driver of CPV-II formation, HMA (or amantadine) treatment would be expected to inhibit CPV-II, but not CPV-I formation. This should be tested and the data included in the study, or if the results are negative, discussed.

*As we have mentioned in the manuscript, we are only testing the role of HMA in reducing virus titer. We agree that there is a possibility that CPV-II formation may have been affected. However, we are not proposing that the HMA is causing a reduction in CPV-II formation or any other activity. We do not think this experiment is required for this manuscript. We are planning to do this in a future study.*

2) The electron microscope data from Fig. 6 should be quantified (e.g. total number of CPV-I and CPV-II per number of fields) for each virus/mutant.

*We thank the reviewer for this suggestion. However, since we do not have the 3D information, the accurate depiction of the total number of CPV-IIs in a cell would require cryo-electron tomography studies, which are out of the scope of this paper. Our conclusions are based on the presence and absence of CPV-II in approximately 60 images collected for each mutant.*

3) Given that several of the constructs show delays in processing, the authors may want to soften some of their language ruling out any effect of processing kinetics on their phenotypes, or more clearly lay out why this is not an issue.

*We have made changes in the revised manuscript to indicate that only E3-E2 cleavage is affected as the furin cleavage requires the trafficking of pE2 to the Golgi.*

**Part III – Minor Issues: Editorial and Data Presentation Modifications**

Please use this section for editorial suggestions as well as relatively minor modifications of existing data that would enhance clarity.

Reviewer #1: (No Response)

Reviewer #2: A thorough discussion of the sequential/structural differences between 6K proteins from different alphaviruses

*We have incorporated a new figure to show the sequence variation among alphaviruses. (Fig. S1)*

Comparison of the roles of M2/Vpu/p7 in the respective viral life cycles

*We have added this information in the revised manuscript.*

Reviewer #3: a) The authors should consider moving Supplemental Table 2 into Figure 1 to make the data more accessible to the reader.

*Modified figure by merging the supplementary table to figure 1.*

b) For Fig. 2A, please clarify which fluorophore (green vs red) is used for E2 vs CP to make it easier for non-expert readers to follow.

*Modified the labels and figure legend.*

c) For Fig. 2c, representative flow plots should be included as supplemental data.

*Added a new flow supplementary figure with a representative flow plot for each mutant (Fig. S2).*

d) Please check the label on the image for Fig. 3B for accuracy. Based on the text it should be CP-E3-mCherryE2-E1, but on the image it is CP-E3-mCherryE2-6K/TF-E1.

*We thank the reviewer for noticing this error on our part. We have now fixed the labels and the figure legend.*

e) When discussing ion channel inhibitors as antivirals, please include discussion of reference 77 (Dey, et al), since that study evaluated amantadine's effects on chikungunya virus.

*We have added the reference and discussed this paper.*

**References**

1.	Chen L, Wang M, Zhu D, Sun Z, Ma J, Wang J, et al. Implication for alphavirus host-cell entry and assembly indicated by a 3.5Å resolution cryo-EM structure. Nat Commun. 2018 Dec 14;9(1):5326.

2.	Ivanova L, Lustig S, Schlesinger MJ. A pseudo-revertant of a Sindbis virus 6K protein mutant, which corrects for aberrant particle formation, contains two new mutations that map to the ectodomain of the E2 glycoprotein. Virology. 1995 Feb 1;206(2):1027–34.

3.	Snyder AJ, Sokoloski KJ, Mukhopadhyay S. Mutating conserved cysteines in the alphavirus e2 glycoprotein causes virus-specific assembly defects. J Virol. 2012 Mar;86(6):3100–11.

4.	Lobigs M, Garoff H. Fusion function of the Semliki Forest virus spike is activated by proteolytic cleavage of the envelope glycoprotein precursor p62. J Virol. 1990 Mar;64(3):1233–40.

5.	Strauss JH, Strauss EG. The alphaviruses: gene expression, replication, and evolution. Microbiol Rev. 1994 Sep;58(3):491–562.

6.	Phinney BS, Blackburn K, Brown DT. The Surface Conformation of Sindbis Virus Glycoproteins E1 and E2 at Neutral and Low pH, as Determined by Mass Spectrometry-Based Mapping. J Virol. 2000 Jun;74(12):5667–78.

7.	Jose J, Snyder JE, Kuhn RJ. A structural and functional perspective of alphavirus replication and assembly. Future Microbiol. 2009 Sep;4(7):837–56.

8.	Jose J, Tang J, Taylor AB, Baker TS, Kuhn RJ. Fluorescent Protein-Tagged Sindbis Virus E2 Glycoprotein Allows Single Particle Analysis of Virus Budding from Live Cells. Viruses. 2015 Nov 27;7(12):6182–99.

9.	Snyder JE, Azizgolshani O, Wu B, He Y, Lee AC, Jose J, et al. Rescue of infectious particles from preassembled alphavirus nucleocapsid cores. J Virol. 2011 Jun;85(12):5773–81.

10.	Jose J, Przybyla L, Edwards TJ, Perera R, Burgner JW, Kuhn RJ. Interactions of the cytoplasmic domain of Sindbis virus E2 with nucleocapsid cores promote alphavirus budding. J Virol. 2012 Mar;86(5):2585–99.

11.	Akahata W, Yang ZY, Andersen H, Sun S, Holdaway HA, Kong WP, et al. A virus-like particle vaccine for epidemic Chikungunya virus protects nonhuman primates against infection. Nat Med. 2010 Mar;16(3):334–8.

12.	Jose J, Taylor AB, Kuhn RJ. Spatial and Temporal Analysis of Alphavirus Replication and Assembly in Mammalian and Mosquito Cells. mBio. 2017 Feb 14;8(1):e02294-16.

13.	Soonsawad P, Xing L, Milla E, Espinoza JM, Kawano M, Marko M, et al. Structural evidence of glycoprotein assembly in cellular membrane compartments prior to Alphavirus budding. J Virol. 2010 Nov;84(21):11145–51.

14.	Liljeström P, Lusa S, Huylebroeck D, Garoff H. In vitro mutagenesis of a full-length cDNA clone of Semliki Forest virus: the small 6,000-molecular-weight membrane protein modulates virus release. J Virol. 1991 Aug;65(8):4107–13.

15.	McInerney GM, Smit JM, Liljeström P, Wilschut J. Semliki Forest virus produced in the absence of the 6K protein has an altered spike structure as revealed by decreased membrane fusion capacity. Virology. 2004 Aug 1;325(2):200–6.

16.	Sanz MA, Carrasco L. Sindbis Virus Variant with a Deletion in the 6K Gene Shows Defects in Glycoprotein Processing and Trafficking: Lack of Complementation by a Wild-Type 6K Gene in trans. J Virol. 2001 Aug;75(16):7778–84.

17.	Warrier R, Linger BR, Golden BL, Kuhn RJ. Role of sindbis virus capsid protein region II in nucleocapsid core assembly and encapsidation of genomic RNA. J Virol. 2008 May;82(9):4461–70.

18.	Firth AE, Chung BY, Fleeton MN, Atkins JF. Discovery of frameshifting in Alphavirus 6K resolves a 20-year enigma. Virol J. 2008 Sep 26;5:108.

19.	Chmielewski D, Schmid MF, Simmons G, Jin J, Chiu W. Chikungunya virus assembly and budding visualized in situ using cryogenic electron tomography. Nat Microbiol. 2022 Aug;7(8):1270–9.
